# Post-mortem brain pathology is related to declining respiratory function in community-dwelling older adults

**DOI:** 10.3389/fnagi.2015.00197

**Published:** 2015-10-21

**Authors:** Aron S. Buchman, Lei Yu, Robert S. Wilson, Robert J. Dawe, Veronique VanderHorst, Julie A. Schneider, David A. Bennett

**Affiliations:** ^1^Rush Alzheimer’s Disease Center, Rush University Medical CenterChicago, IL, USA; ^2^Department of Neurological Sciences, Rush University Medical CenterChicago, IL, USA; ^3^Department of Behavioral Sciences, Rush University Medical CenterChicago, IL, USA; ^4^Department of Diagnostic Radiology and Nuclear Medicine, Rush University Medical CenterChicago, IL, USA; ^5^Department of Neurology, Beth Israel Deaconess Medical CenterBoston, MA, USA; ^6^Harvard Medical SchoolBoston, MA, USA; ^7^Department of Pathology (Neuropathology), Rush University Medical CenterChicago, IL, USA

**Keywords:** aging, respiratory decline, respiratory muscles, spirometry, Alzheimer’s disease pathology, nigral neuronal loss, macroscopic infarct, neuropathology

## Abstract

Damage to brain structures which constitute the distributed neural network that integrates respiratory muscle and pulmonary functions, can impair adequate ventilation and its volitional control. We tested the hypothesis that the level of brain pathology in older adults is associated with declining respiratory function measured during life. 1,409 older adults had annual testing with spirometry (SPI) and respiratory muscle strength (RMS) based on maximal inspiratory and maximal expiratory pressures (MEPs). Those who died underwent structured brain autopsy. On average, during 5 years of follow-up, SPI and RMS showed progressive decline which was moderately correlated (*ρ* = 0.57, *p* < 0.001). Among decedents (*N* = 447), indices of brain neuropathologies showed differential associations with declining SPI and RMS. Nigral neuronal loss was associated with the person-specific decline in SPI (Estimate, −0.016 unit/year, S.E. 0.006, *p* = 0.009) and reduction of the slope variance was equal to 4%. By contrast, Alzheimer’s disease (AD) pathology (Estimate, −0.030 unit/year, S.E. 0.009, *p* < 0.001) and macroscopic infarcts (−0.033 unit/year, S.E., 0.011, *p* = 0.003) were associated with the person-specific decline in RMS and reduction of the slope variance was equal to 7%. These results suggest that brain pathology is associated with the rate of declining respiratory function in older adults.

## Introduction

To maintain adequate ventilation, a neural network integrates respiratory muscle and pulmonary functions (Laviolette et al., 2013; Butler et al., 2014; Richter and Smith, 2014). This distributed network begins in the brain and extends to the spinal cord where peripheral nerves exit to innervate and activate respiratory muscles in the periphery (Laviolette et al., 2013; Butler et al., 2014; Richter and Smith, 2014). Damage to brain structures which constitute this distributed network may impair respiration separate from direct effects on the respiratory muscles and intrinsic lung tissues. Furthermore, respiration is also under volitional brain control. Recent work in this cohort and by others has shown that brain pathology is associated with the progressive loss of motor function in older adults (Gorelick et al., 2011; Jack et al., 2011; Stern et al., 2012; Boyle et al., 2013; Buchman et al., 2014). It is unknown, if brain pathology is also associated with declining respiratory function in older adults.

We tested the hypothesis that the level of brain pathology is associated with the rate of declining respiratory function in older adults. We used data from 1,409 older persons participating in a community-based cohort study that included annual structured clinical exam and autopsy at death (Bennett et al., 2012). Respiratory function was summarized using two composite measures developed in a series of prior publications (Buchman et al., 2008a,b, 2009; Boyle et al., 2009). Composite spirometry (SPI) was derived from three spirometric measures (vital capacity, VC; forced expiratory volume in 1 second, FEV1; and peak expiratory flow, PEF). Composite respiratory muscle strength (RMS) was based on maximal inspiratory pressures (MIPs) and maximal expiratory pressures (MEPs). Post-mortem indices of nine age-related brain pathologies were collected at brain autopsy.

While SPI can be used to characterize restrictive or obstructive lung disease, SPI will also vary with RMS. Moreover, the central nervous system respiratory network integrates both intrinsic lung function and RMS to maintain ventilation. Therefore, we employed modeling which would account for the correlated function of spirometric measures and RMS. Bivariate random coefficient models were used to estimate simultaneously the rates of change in composite measures of SPI and RMS. Then, using data from decedents, we tested whether indices of degenerative brain pathologies were associated with the rates of change of SPI and RMS.

## Materials and Methods

### Participants

Participants are from the Rush Memory and Aging Project, a community-based cohort study which began in Bennett et al. (2012). The study was approved by the Institutional Review Board of Rush University Medical Center. Written informed consent and an anatomic gift act for brain donation at the time of death was obtained from all study participants.

A completed baseline with respiratory testing has been obtained in 1,754 participants examined since it was added in 2001. This study excluded 345 individuals lacking a 2nd evaluation since it focuses on change in respiratory function. These included individuals who died before their 2nd visit, had not been in the study long enough for follow-up, or valid measures were not available. The 1,409 included in these analyses had an average of 5 follow-up assessments (mean = 5.0, SD = 3.4). Baseline age, education, SPI and RMS testing were similar in participants with 5 or less follow-ups vs. those with more than 5 (results not shown), but there were more males with 5 or less follow-ups (27.7% vs. 22.2%, Chi-square = 5.0863, df = 1, *p* = 0.024).

### Assessment of Respiratory Function

An annual uniform structured evaluation included medical history and clinical examination by trained registered nurses (Bennett et al., 2012).

SPI: two trials obtained with a hand-held spirometer which measured VC, FEV1 and PEF (MicroPlus Spirometer MS03, MicroMedical Ltd. Kent, UK). A composite SPI score was based on the average of the *z* scores for VC, FEV1 and PEF as described in prior publications Buchman et al. (2008a,b, 2009) and Boyle et al. (2009).

RMS: muscles needed for adequate respiration include the diaphragm and intercostal muscles which are innervated by cervical and thoracic root segments not involved in limb movements. One can isolate and estimate RMS by measuring the maximal pressures generated during isometric inspiration and expiration (Kim and Sapienza, 2005). A hand-held device that contains a pressure sensitive transducer was used to assess MIP and MEP in cm H_2_O (MicroMouth Pressure Meter MP01; MicroMedical Ltd., Kent, UK). Two trials of both were obtained. A composite RMS score was computed from the average of the *z* scores for MIPs and MEPs (Buchman et al., 2008a,b, 2009; Boyle et al., 2009). Since self-report pulmonary disease history was not collected in MAP, possible pulmonary disease was considered if the ratio of FEV 1 /FVC was <0.7, as suggested by previous literature (Iqbal et al., 2002).

### Comorbidities and Other Covariates

Age at enrollment, sex and years of education were recorded at the baseline interview. Seven chronic diseases were documented at baseline and each follow-up visit based on self-report of hypertension, diabetes, myocardial infarction, cancer, thyroid disease, head trauma, stroke and smoking status. Respiration could be affected in participants who were receiving one or more medications used to treat chronic pulmonary diseases including anticholinergics, α-adrenergics, theophylline, steroid inhalants, and leukotrienes; medications for Alzheimer’s disease (AD) including central acetylcholinesterase inhibitors (e.g., donepezil), NMDA receptor blockers (e.g., memantine), parasympathomimetic agents (e.g., rivastigmine), alkaloid (e.g., galantamine) or medications for Parkinson’s disease (PD) including levodopa or dopaminergic agonists, anticholinergics, monoamine oxidase inhibitor (e.g., rasagiline), catechol-O-Methyltransferase inhibitor (e.g., entacapone), NMDA receptor antagonist (e.g., amantadine). Medications were inspected and coded using the Medi-Span system (Medi-Span, Inc.; Buchman et al., 2008b).

### Post-Mortem Indices

Brain removal, tissue sectioning and preservation, and a uniform gross and microscopic examination with quantification of post-mortem indices followed a standard protocol (Bennett et al., 2012). Nine post-mortem indices were examined.

Indices of cerebrovascular disease (CVD) pathologies which assessed parenchymal and cerebral vessel pathology were collected. We assessed the presence of macroscopic infarcts. We reviewed 1 cm slabs and recorded the age, volume (in mm^3^), side, and location of all cerebral infarcts visible to the naked eye as previously reported (Schneider et al., 2003). Hemorrhagic infarcts were included in analyses. There was no minimum size required for macroscopic infarcts. All grossly visualized and suspected macroscopic infarcts were microscopically reviewed for histologic confirmation. Infarct age (acute, subacute and chronic) was estimated by gross histologic features and degree of cavitation. In all cases we examined for the presence of microinfarcts: (Arvanitakis et al., 2011). The following regions were dissected, processed and embedded for review: middle frontal cortex middle temporal cortex, anterior cingulate cortex, inferior parietal cortex, entorhinal cortex, hippocampus, anterior basal ganglia, anterior thalamus, and hemisection of midbrain including substantia nigra. Hematoxylin and eosin stained six micron sections were used to identify microscopic infarcts. Microscopic infarcts were defined as any infarct seen only by microscopic examination. Microscopic infarcts ranged from cavitated to a focal area of shrinkage due to astrogliosis associated with few remaining macrophages. Any chronic microinfarct visualized microscopically by the neuropathologist was included in our analyses. The quantity of macroinfarcts and microinfarcts, were summarized using a two level predictor measure: as absent or present as previously described in Schneider et al. (2003) and Arvanitakis et al. (2011).

Assessment for cerebral vessel pathology was also systematically conducted, including for large and small vessels. Atherosclerosis in the circle of Willis was assessed on gross examination. Severity was graded on a scale from 0 (no atherosclerosis) to 6 (severe atherosclerosis, with all visualized arteries affected or one artery completely occluded).

Arteriolosclerosis was documented on H&E stained sections of the anterior basal ganglia (caudate, putamen, globus pallidus, and internal capsule) (10). Severity of this pathology was also graded, and ranged from 0 (no arteriolosclerosis) to 6 (severe or complete small vessel occlusion). For the purpose of analyses for this study, severity of both these vessel pathologies were grouped into four levels: not present, mild, moderate, and severe.

Cerebral amyloid angiopathy (CAA) was determined on immunohistochemical examination of meningeal and parenchymal vessels in five neocortical regions (midfrontal, middle temporal, angular and calcarine cortices). Slides were cut from paraffin embedded blocks and stained with one of three antibodies to amyloid beta [10D5 Beta Amyloid, 17–24 (4G8) Covance Madison, 1:9000; Anti-Human Amyloid-Beta 1–16 (10D5) Elan Pharmaceuticals, San Francisco, 1:300; Anti-Human Beta-Amyloid, (6F/3D), DAKO North America, Carpinteria, 1:50]. For each region, vascular deposition of amyloid beta was scored from 0 (no deposition) to 4 (circumferential deposition more than 75% of the region). The maximum score between the meningeal and parenchymal amyloid angiopathy scores was used as the amyloid angiopathy pathology score for that region. Scores were then averaged across the five regions. We used this overall average score to develop a four level rating scale, based on the data distribution and pathology severity at the different levels as determined by the neuropathologist. For analyses in this study, we used amyloid angiopathy severity data categorized as none, mild, moderate, and severe grades.

AD pathology was assessed in the frontal, temporal, parietal, and entorhinal cortex, and the hippocampus, as previously described in Bennett et al. (2006). Bielschowsky silver stain was used to visualize neuritic plaques, diffuse plaques, and neurofibrillary tangles. Briefly, the operator used a graticule to project a grid to count numbers of each pathological marker in a 1 mm^2^ area (magnification ×100) under the microscope. Counts were performed by a board-certified neuropathologist or trained technician blinded to all clinical data. Plaques and tangle counts had different ranges and were not normally distributed; therefore, we created standardized scores for each plaque and tangle count in each cortical area as previously described. These scaled scores for each region were then averaged across the five regions (midfrontal, superior temporal, inferior parietal, entorhinal, and hippocampal cortex) to develop summary scores for diffuse plaques, neuritic plaques, and neurofibrillary tangles for each subject. We then averaged the summary scores of the three AD markers from the five sites examined to yield the global measure of AD pathology for each subject used in these analyses (Bennett et al., 2006).

Transactive response DNA-binding protein 43 (TDP-43) was assessed in six brain regions (amygdala, entorhinal cortex, CA1/subculum, dentate gyrus, middle temporal cortex, midfrontal cortex) with a monoclonal antibody to phosphorylated TDP-43 (pS409/410; 1:100; Neumann et al., 2009). In each region, neuronal and glial TDP-43 cytoplasmic inclusions were rated on a six-point scale from none to severe, and regional ratings were averaged to yield a total score (Wilson et al., 2013).

PD pathology was based on the assessment of nigral neuronal loss and the presence of Lewy body pathology. Dissection of diagnostic blocks included a hemisection of midbrain which included substantia nigra. Nigral neuronal loss was assessed in the substantia nigra in the mid to rostral midbrain near or at the exit of the 3rd nerve using H&E stain and 6 micron sections using a semi-quantitative scale (0–3) was employed and used in these analyses (Buchman et al., 2012). Lewy body disease pathology was identified with antibodies to alpha-synuclein using alkaline phosphatase as the chromogen. The presence or absence of Lewy body pathology was based on assessment of six brain regions including substantia nigra, limbic cortex, and several neocortical regions as previously described. A four level semiquantitative measure for nigral neuronal loss was employed (Buchman et al., 2012).

### Statistical Analyses

Pairwise associations of baseline SPI and RMS with demographic variables were examined using Pearson correlations. Declining SPI and RMS occur simultaneously in the same individuals and since they are controlled by a common network and share volitional brain control they are likely to manifest correlated function. Therefore, we employed bivariate random coefficient models which used the repeated clinical observations to estimate the correlation structure between SPI and RMS. This single model examines the associations of baseline level of each outcome with their rates of change and the extent to which simultaneous change in both of these outcomes are associated. We elected not to include quadratic change because both fit statistics of Akaike’s Information Criterion (AIC) and Bayesian Information Criterion (BIC) suggest that linear change was sufficient. In addition, assessment of plots of a random sample of participants did not show evidence of non-linearity.

Bivariate random coefficient models which adjusted for age, sex and education were used to estimate simultaneously the levels and rates of change of SPI and RMS. The correlation of level and change in SPI and RMS was estimated from a joint distribution of the random effects.

Using similar analyses in decedents, we added terms for postmortem indices and estimated to what extent post-mortem indices alone and together were associated with the trajectories of SPI and RMS during life. Models were examined graphically and analytically and assumptions were judged to be adequately met. *A priori* level of statistical significance was 0.05. Programing was done in SAS version 9.3 (SAS Institute Inc, Cary, NC, USA; SAS Institute Inc, 2002–2003).

## Results

### Descriptive Properties of Respiratory Function in Older Adults

Clinical characteristics of participants included in these analyses are in Table 1.

**Table 1:** **Clinical characteristics of participants in these analyses**.

Variable	All (*N* = 1409)	Alive (*N* = 843)	Deceased (*N* = 566)
	**Mean (SD) Or *N* (%)**	**Mean (SD) Or *N* (%)**	**Mean (SD) Or *N* (%)**	∣rule
**Demographics**
Age at baseline (years)	80.1 (7.60)	77.8 (7.77)	83.6 (5.79)
Age at last visit/death (years)	85.1 (7.76)	83.3 (8.14)	89.6 (6.07)
Sex (female)	1047 (74.3%)	664 (78.8%)	383 (67.7%)
Education (years)	14.6 (3.17)	14.7 (3.32)	14.4 (2.92)
**Clinical diagnoses**
No cognitive impairment (NCI)	1012 (71.8%)	666 (79.0%)	346 (61.1%)
Mild cognitive impairment (MCI)	335 (23.8%)	163 (19.3%)	172 (30.4%)
AD dementia	62 (4.4%)	14 (1.7%)	48 (8.5%)
Parkinson’s disease	21 (1.5%)	6 (0.7%)	15 (1.6%)
**Pulmonary function tests**
Forced expiratory volume (L)	1.7 (0.56)	1.8 (0.55)	1.5 (0.54)
Vital capacity (L)	1.9 (0.62)	2.0 (0.61	1.8 (0.61)
Peak expiratory flow (L/min)	266.7 (111.64)	286.8 (109.45)	236.9 (108.27)
FEV/VC	0.9 (0.09)	0.9 (0.08)	0.8 (0.10)
Maximal inspiratory pressure (mm H_2_O)	41.7 (20.61)	45.2 (21.15)	36.3 (18.57)
Maximal expiratory pressure (mm H_2_O)	67.1 (24.57)	70.0 (24.86)	62.8 (23.49)
COPD (FEV/VC <0.7)	76 (5.4%)	26 (3.1%)	50 (8.8%)
**Self-report medical conditions**	1.4 (1.06)	1.4 (1.01)	1.6 (1.12)
Hypertension	757 (53.73%)	443 (52.6%)	314 (55.5%)
Diabetes	181 (12.85%)	102 (12.1%)	79 (14.0%)
Myocardial infarction	151 (10.73%)	61 (7.25%)	90 (15.9%)
Cancer	440 (31.23%)	263 (31.2%)	177 (31.3%)
Thyroid disorder	278 (19.74%)	168 (20.0%)	110 (19.4%)
Head trauma	88 (6.25%)	53 (6.29%)	35 (6.19%)
Stroke	132 (10.32%)	56 (7.73%)	76 (13.7%)
**Post-mortem indices (*N* = 447)**
Post-mortem interval (hrs)			8.35 (7.41)
Chronic macroinfarct (1 or more)			167 (37.4%)
Chronic microinfarct (1 or more)			138 (30.9%)
Atherosclerosis (moderate-severe)			159 (35.7%)
Arteriolosclerosis (moderate-severe)			158 (35.5%)
Cerebral amyloid angiopathy			152 (34.2%)
Alzheimer’s disease (based on NIA reagan)			285 (63.8%)
TDP-43			220 (53.5%)
Lewy body disease present			100 (22.4%)
Nigral neuronal loss (moderate-severe)			55 (12.3%)

SPI at baseline ranged from −2.35 to 3.20 with a more positive value indicative of better function, on average SPI was 0.02 (SD = 0.92). SPI was associated with age (*r* = −0.32; *p* < 0.001) and education (*r* = 0.23, *p* < 0.001). At baseline, SPI was higher in men (*t*_489_ = −20.14, *p* < 0.001).

RMS at baseline ranged from −2.13 to 3.41 with a more positive value indicative of better strength, on average respiratory function was 0.01 (SD = 0.91). RMS was associated with age (*r* = −0.28; *p* < 0.001) and education (*r* = 0.13, *p* < 0.001). At baseline, RMS was higher in men (*t*_537_ = −15.97, *p* < 0.001).

### Rates of Change in Spirometry and Respiratory Muscle Strength in Older Adults

**Figure 1:**
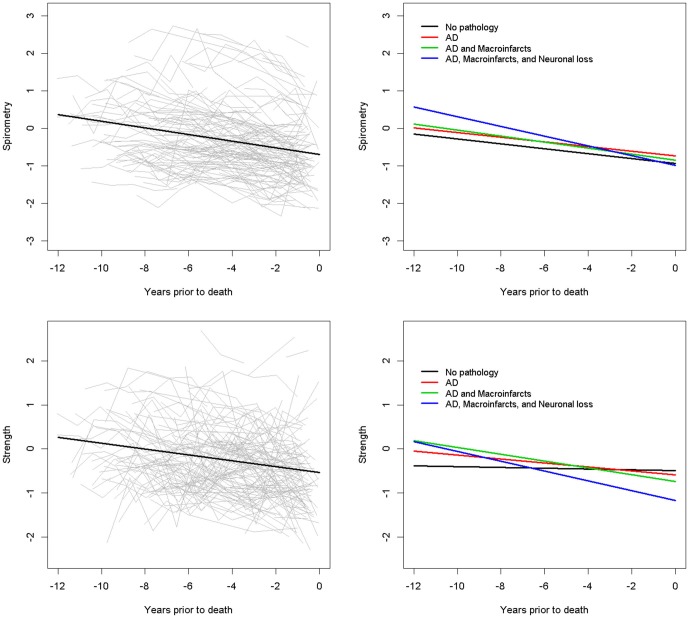
**Change in spirometry (SPI) and respiratory muscle strength (RMS) and the effect of more brain pathology on their rates of change.** The left panels show change in SPI (top) and RMS (bottom) during the study. Crude paths of change (gray lines) and mean paths of change predicted by the model (black lines) in SPI (top) and RMS (bottom). To facilitate visualization data from a 25% random sample of decedents is illustrated in the left panels. To display the association of brain pathology on the rate of change in respiration, four hypothetical average participants with their estimated rate of declining respiration based on the model which included all the cases analyzed in this study are illustrated. The right panels show the model derived predicted paths of SPI (top) and RMS (bottom) for four participants with increasing burden of brain pathology: (1) Black line, the predicted path for a participant with No pathology; (2) Red line, the predicted path for a participant with Alzheimer’s disease (AD) pathology; (3) Green line, the predicted path for a participant with AD pathology and macroinfarcts; (4) Blue line, the predicted path for a participant with AD pathology, macroinfarcts and severe nigral neuronal loss.

During up to 13 years of follow-up, both SPI (Estimate −0.068, S.E. 0.002, *p* < 0.001) and RMS (Estimate −0.050, S.E. 0.002, *p* < 0.001) declined. Observed paths of change (gray lines) and model estimated mean paths of change (black) in SPI (top) and RMS (bottom) are illustrated on the left panel of Figure 1.

**Figure 2:**
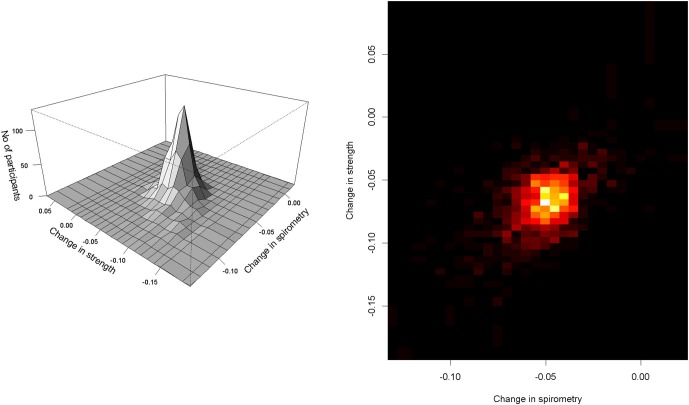
**Annual rates of change in SPI and RMS.** On the left is a two-dimensional histogram of annual rates of change in SPI and RMS estimated by simultaneous random effects model (Table 2). The figure on the right depicts the density of the number of participants shown in the two dimensional histogram with yellow showing increased density compared to the shades of red. *X* axis shows change in SPI and *Y* axis shows the change in RMS. In both portions of the figure, each point illustrates the person-specific change in both aspects of respiratory function. As can be seen on the right, nearly all values are less than zero, for SPI and RMS since both declined in nearly all cases.

Person-specific rates of change of SPI and RMS was moderately correlated (Table 2). Figure 2 is a two-dimensional histogram of the model derived annual rates of change in SPI and RMS (left), and the corresponding density map (right). The figures highlight the relationship between declining SPI and RMS.

**Table 2:** **Correlation of baseline and longitudinal changes in spirometry and respiratory muscle strength**.

Variable	Respiratory muscle strength_bl_	Change in spirometry	Change in respiratory muscle strength
Spirometry_bl_	0.63*	−0.26*	−0.130
Respiratory	–	0.05	−0.25*
Muscle strength_bl_
Change in spirometry		–	0.57*

Estimated correlation (*p < 0.002) between of the baseline and longitudinal terms included in the simultaneous bivariate random coefficient models (Figure 2). The core model included terms for baseline spirometry and respiratory muscle function and their interaction with Time i.e., the rate of change in spirometry and the rate of change in and respiratory muscle function.

The rates of change remained associated when adjusting for demographics (*r* = 0.49, *p* < 0.001). In these models, increasing age and male sex, but not education were associated with more rapid decline in SPI and RMS (*p*’s < 0.001).

In further analyses, we examined each of the individual measures used to construct these composite measures. Each of the individual measures declined and showed similar associations with age and sex (Table 3).

**Table 3:** **Linear-mixed effect models showing the effect of demographic variables on the rate of change of spirometric measures, MIPs and MEPs in community-dwelling older adults**.

	Spirometry			Respiratory muscle strength	
Model term	PVC	FEV	PEF	MEP	MIP
Annual rate of change	−0.069	−0.066	−0.052	−0.046	−0.043
	0.003, <0.001	0.003, <0.001	0.003, <0.001	0.003, <0.001	0.003, <0.001
Age × annual rate of change	−0.001	−0.001	−0.001	−0.001	−0.001
	0.0003, <0.001	0.0003, <0.001	0.0003, 0.005	0.0004, <0.001	0.0004. 0.018
Sex × annual rate of change	−0.021	−0.025	−0.022	−0.023	−0.020
	0.006, <0.001	0.005, <0.001	0.006, <0.001	0.007, <0.001	0.006, 0.001
Education × annual rate of change	−0.001	−0.001	−0.0003	−0.0005	0.001
	0.001, 0.189	0.001, 0.061	0.009, 0.700	0.001, 0.613	0.001, 0.195

*Linear-mixed effect models were performed for each of the 3 components (vital capacity, forced expiratory volume, peak expiratory force) used to construct composite spirometry and the two components used to construct composite respiratory muscle strength (RMS; maximal expiratory pressure, MEP and maximal inspiratory pressure, MIP). Each model included a term for Time in years since the baseline which quantifies the rate of change in the spirometry or RMS measure which was being examined. To control for the effect of demographic variables, we also included terms for age, sex and education and their interaction with the annual rate of change in the respiratory measure which was examined. The cross sectional terms which were included in these models are not shown in this table. The mean annual rate of change in standardized units for spirometric measures was larger than for RMS measures [PVC: −0.07 (SD = 0.02); FEV: −0.07 (SD = 0.02); PEF: −0.06 (SD = 0.02); MEP: −0.05 (SD = 0.02); MIP: −0.05 (SD = 0.02)]*.

Poor health and smoking can affect respiration. Controlling for the number of chronic health conditions present at baseline and a history of present or past smoking did not account for the correlation of the rate of change in SPI and RMS (*r* = 0.51, *p* < 0.001).

Level of physical activity might also affect respiration. The correlation of the rate of change in SPI and RMS was unchanged when we controlled for self-reported physical activity (*r* = 0.49, *p* < 0.001).

The use of pulmonary medications could affect respiration. Controlling for pulmonary medications did not attenuate the correlation of declining SPI and RMS (*r* = 0.49, *p* < 0.001). Adjustment for treatment with AD or PD medications did not affect the correlation of declining SPI and RMS (AD: *r* = 0.49, *p* < 0.001; PD: *r* = 0.48, *p* < 0.001).

In a series of sensitivity analyses, the rates of change in SPI and RMS remained strongly associated when we excluded cases with clinical dementia at baseline (*N* = 62, 4.4%; *r* = 0.46, *p* < 0.001); PD (*N* = 21, 1.5%; *r* = 0.50, *p* < 0.001) and stroke (*N* = 132, 10.3%; *r* = 0.46, *p* < 0.001).

### Brain Pathology and Rates of Change in Spirometry and Respiratory Muscle Strength in Older Adults

Since respiration is controlled by a distributed CNS network and is subject to volitional control, we examined whether indices of brain pathologies were associated with the rates of change in SPI and RMS. There were 566 cases (31.8%) who died during the study and 478 (84.5%) underwent a uniform structured brain autopsy which has been completed in the first 447 consecutive individuals. A summary of the baseline clinical characteristics of those who died vs. those who did not die and post-mortem indices are included in Table 1. At their last visit, about 40% of those undergoing autopsies had clinical dementia [AD (*N* = 154, 34.7%; vascular dementia *N* = 10, 2.3%; Lewy body dementia *N* = 7; 1.6%; other *N* = 4, 1.0%)]. The average participant had three different pathologies; almost all (>97%) had 1 or more pathologies (1 = 13.7%; *2* = 19.2%; *3* = 20.8%; *4* = 22.4%; *5* = 13.2%, 6 or more 7.8%). Older age at death was associated with the presence of more brain pathologies (*r* = 0.19, *p* < 0.001).

First, we examined if SPI and RMS showed correlated change in participants who died. We repeated the core model (Table 2) and adjusted for demographics in deceased participants who underwent autopsy. The simultaneous change in lung function and RMS was strongly related (*r* = 0.61, *p* < 0.001). The rates of declining SPI and RMS was more rapid in males (*p*’s < 0.02), but did not vary with age or education.

Next, we compared the rates of change between SPI and RMS between participants who died and those who remained living. Those who died showed more rapid declining SPI (Estimate −0.024, S.E., 0.005, *p* < 0.001) and RMS (Estimate −0.018, S.E., 0.005, *p* = 0.001). While the rate of respiratory decline was about 40% more rapid in those who died compared to those who did not die, the correlated change between the rates of declining SPI and RMS remained significant in both groups (*r* = 0.45, *p* < 0.001). To determine if brain neuropathologies were associated with declining SPI and RMS, in separate models, we added terms for each of the 9 post-mortem indices as well as their interaction with time to the core model described above (Table 4). Three of the nine pathologies (nigral neuronal loss, AD pathology and macroinfarcts) remained associated with the rate of change of either SPI or RMS when included together with the other pathologies.

**Table 4:** **Associations of individual brain pathologies and the annual rate of change in spirometry and respiratory muscle strength**.

Model	Pathology	Pathology × annual rate of change in spirometry Estimate (S.E., *p*-Value)	Pathology × annual rate of change in respiratory muscle strength Estimate (S.E., *p*-Value)
1	Macroinfarcts	−0.018	−0.030
		(0.010, 0.088)	(0.011, 0.009)
2	Microinfarcts	−0.006	−0.025
		0.010, 0.566	0.011, 0.030
3	Alzheimer disease	0.003	−0.029
		0.008, 0.668	0.009, 0.002
4	Lewy body disease	−0.024	−0.014
		0.012, 0.037	0.013, 0.269
5	Nigral neuronal loss	−0.017	−0.011
		0.006, 0.005	0.007, 0.093
6	Atherosclerosis	−0.009	−0.005
		0.006, 0.129	0.006, 0.462
7	Arteriolosclerosis	−007	−007
		0.006, 0.195	0.006, 0.262
8	Cerebral amyloid angiopathy	−0.008	−0.010
		0.005, 0.117	0.006, 0.061
9	TDP-43	−0.005	−0.004
		0.005, 0.299	0.005, 0.478

*Each row shows the results estimated from simultaneous bivariate random coefficient models which included terms for baseline and rate of change in spirometry (SPI) and RMS. Each of the nine models included terms to control for demographics (age, sex, education) and for each brain pathology alone. This table only shows the interaction between brain pathologies and the rates of change in spirometry, RMS and the correlation between the rates of change in spirometry and RMS. The other terms (age, sex and education) included in the model and the other interaction terms are not shown*.

Brain pathologies were differentially associated with declining respiratory function. Nigral neuronal loss was strongly associated with the rate of change in SPI and there was a marginal association for Lewy body pathology and macroinfarcts. The presence of macroinfarcts and AD pathology were independently associated with the rate of change in RMS and there was a marginal association for nigral neuronal loss (Table 5).

**Table 5:** **Brain pathologies independently associated with the annual rate of change in spirometry and respiratory muscle strength prior to death**.

(A)	Associations of brain pathologies with annual rate of change in spirometry (SPI)		Associations of brain pathologies with annual rate of change in respiratory muscle strength (RMS)	
	Term	Estimate (S.E., *p*-value)	Term	Estimate (S.E., *p*-value)
	Macroinfarct ×	−0.018	Macroinfarct ×	−0.033
	annual rate of change in SPI	(0.010, 0.075)	annual rate of change in RMS	(0.011, 0.003)
	AD pathology ×	0.003	AD pathology ×	−0.030
	annual rate of change in SPI	(0.010, *p* = 0.718)	annual rate of change in RMS	(0.011, *p* < 0.001)
	Nigral neuronal loss ×	−0.016	Nigral neuronal loss ×	−0.011
	annual rate of change in SPI	(0.006, *p* = 0.009)	annual rate of change in RMS	(0.007, *p* = 0.089)
**(B)**	**Interaction of brain pathologies with annual rate of change in spirometry (SPI)**		**Interaction of brain pathologies with annual rate of change in respiratory muscle strength (RMS)**	
	**Term**	**Estimate (S.E., *p*-value)**	**Term**	**Estimate (S.E., *p*-value)**
	Macroinfarct ×	−0.019	Macroinfarct ×	−0.033
	annual rate of change in SPI	(0.010, *p* = 0.072)	annual rate of change in RMS	(0.011, *p* < 0.003)
	AD pathology ×	0.003	AD pathology ×	−0.030
	annual rate of change in SPI	(0.008, *p* = 0.708)	annual rate of change in RMS	(0.009, *p* < 0.001)
	Lewy body pathology ×	−0.022	Lewy body pathology ×	−0.013
	annual rate of change in SPI	(0.012, *p* = 0.056)	annual rate of change in RMS	(0.013, *p* = 0.313)

*After we reviewed the results of the analyses summarized in Table 4; we examined a model which included the pathologies which showed an association with either the rate of change in spirometry or RMS prior to death to determine which pathologies showed independent associations with declining respiratory function, (A,B) each show the results estimated from a single simultaneous bivariate random coefficient models which included terms for baseline and the annual rates of change in spirometry and RMS (Table 3) which adjusted for age, sex, education and their interaction with annual rates of change (not shown). In addition, these models included terms for the brain pathologies and their interactions with the annual rates of change in spirometry and RMS. This table only shows the interaction between brain pathologies and the annual rates of change in spirometry and RMS. We show models with nigral neuronal loss (A) and LBD pathology (B) separately because we have shown previously that nigral neuronal loss may link LBD pathology with clinical outcomes*.

Given the sex differences observed in the rate of respiratory decline, we examined whether the associations of brain pathologies and declining respiration varied with age or sex by adding three-way interaction terms to the model summarized in Table 5. The associations of these brain pathologies with the rate of change in SPI and RMS did not vary with age or sex (results not shown).

The right panels of Figure 1 illustrate the additive effects of these pathologies on the rate of change of SPI and RMS by showing the trajectories for four average participants with increasing burden of neuropathology. In the model with nigral neuronal loss, reduction of the person-specific SPI slope variance was equal to 4%. In contrast, in the model with AD pathology and macroinfarcts, reduction of the person-specific RMS slope variance was equal to 7% (Table 6).

**Table 6:** **Percentage of the variance of rate of change in spirometry and respiratory muscle strength explained by demographics and post-mortem indices**.

Model term(s)	Percentage of variance of change in spirometry	Percentage of variance of change in respiratory muscle strength
Age, Sex, Education	8.88%	17.02%
Neuropathologies	3.27%	7.37%
Macroinfarcts	NS	6.27%
AD pathology	NS	1.10%
Nigral neuronal loss	3.27%	NS
Total % of variance	12.15%	24.39%

## Discussion

Late-life motor impairment is common and progressive in many older adults with a marked heterogeneity in its rate of progression (Kim and Sapienza, 2005; Rosso et al., 2013). Respiratory function requires the integrated actions of intrinsic lung function and respiratory muscles to maintain adequate ventilation. Cross sectional studies have shown that assessment of respiration with SPI and RMS based on MIPs and MEPs show lower levels with increasing age (Enright et al., 1994; Sclauser Pessoa et al., 2014). While, longitudinal studies have shown that spirometric measures decline in older adults, these studies have not reported concurrent assessments of RMS (Tang et al., 2013, 2014). In prior studies in this cohort, we examined the inter-relationship of concurrent spirometric and RMS measures in older adults at baseline and their association with survival, mobility decline and incident disability (Buchman et al., 2008a,b, 2009). However, our prior studies did not examine the inter-relationship of the rate of change in SPI and RMS measures over time.

The current study extends these prior reports in several important ways. Longitudinal studies of conventional motor performances including gait and use of the extremities, dshow that while most older individuals show some degree of decline over time, some show stable function for many years (Buchman et al., 2014). In the current study, we found that while there is substantial inter-individual heterogeneity (Figure 1), nearly all participants showed some degree of loss of spirometric and RMS during the course of this study. Further work is needed to delineate the mechanisms which underlie the progressive loss of respiratory function.

Recent work in this cohort and by others has shown that the accumulation of degenerative brain changes may contribute to loss of a wide range of cognitive and motor abilities in older adults (Gorelick et al., 2011; Jack et al., 2011; Stern et al., 2012; Boyle et al., 2013; Buchman et al., 2014). While it is well known that damage to brain structures can impair adequate ventilation and its volitional control, we are unaware of prior studies which have examined the relation of post-mortem neuropathology and respiratory function measured during life. The current findings provide novel data showing that brain pathologies are also associated with declining respiratory function. While TDP-43 may affect respiratory function in ALS, in the current study of community-dwelling older adults it was not associated with declining respiration (Pokrishevsky et al., 2012). Other indices of neuropathology were associated with respiratory function, but some were related to declining SPI and others were related to RMS. Nigral neuronal loss was associated with SPI, but not with RMS. Recent reports have described possible lung dysfunction in classical PD (Wang et al., 2014). Nigral neuronal loss can be caused by a wide range of disease processes for example AD, PD and CVD. In the current study Lewy body pathology was only marginally associated with declining SPI. Since Lewy body pathology only occurs in about 20% of cases, while up to 40% of older adults may have some degree of nigral neuronal loss, more cases may be needed to determine whether there is differential association of spiromtey and RMS with Lewy body pathology as well as nigral neuronal loss, linking more directly to PD (Buchman et al., 2012). By contrast, AD pathology and macroinfarcts were associated with RMS but not with SPI. This latter association extends prior reports in this cohort of a link between decreased muscle bulk and RMS in old age with incident mild cognitive impairment (MCI) and AD (Buchman et al., 2005; Boyle et al., 2009; Sanches et al., 2014). The varied associations observed in the current study, underscores the importance of analyzing different components of the respiratory network separately, since these findings would not be apparent if only one aspect is measured.

Degenerative brain changes explained about 4% of the variance of declining SPI and 7% of declining RMS in the current study. The bulk of respiratory decline was unexplained by traditional brain pathologies, suggesting that unidentified brain pathologies, disease processes or structures remain to be identified to explicate the biology underlying respiratory decline in older adults Given the extent of respiratory impairments in old age, even the modest effect sizes observed in the current study are likely to be important. It is worth comparing the current results for declining respiration to cognitive decline which derives exclusively from degenerative changes limited to brain structures. In recent reports which have employed a similar approach for examining the pathologic basis for cognitive decline, AD pathology accounted for about 20% of the variance of declining cognition in older adults. Other pathologies including Lewy body pathology and macroinfarcts accounted for 8% and 3% of the variance of cognitive decline (Boyle et al., 2013; Buchman et al., 2014). The small percentage of variance of respiratory function accounted for by brain pathology is similar to findings for other volitional motor performance whose pathways also extend beyond the brain (Buchman et al., 2014). Since other central nervous system structures and many non-neurologic constituents, e.g., intrinsic lung and muscle tissues, are essential for respiration and other motor performances, it is not surprising that degenerative brain changes only accounts for a minority of the variance of declining respiratory and motor function in older adults. Nonetheless, the current findings lend support to the notion that degenerative brain changes may make a more substantial contribution to morbidity and mortality in old age than previously suspected (James et al., 2014).

The study has strengths that lend confidence in the findings. All subjects were recruited from the community and underwent an annual detailed clinical evaluation. Uniform, structured clinical and post-mortem procedures were followed by examiners blinded to data collected at other visits. Clinical follow-up and autopsy rates were very high. The analytic approach employed in this study permitted simultaneous characterization of both cross sectional and longitudinal association in the same individuals. The availability of post-mortem indices provided an opportunity to examine whether degenerative brain changes are associated with declining respiration in older adults. Like all observational clinical-autopsy studies, these analyses cannot determine if the association with pathology is causal or whether they are affected by a latent variable or whether primary pulmonary diseases could affect brain function. The large number of cases provided sufficient power to evaluate demographic and clinical variables that might have affected results.

This study has important limitations. First, participants were selected by their willingness to participate in these studies and their rates of chronic diseases may be lower than other studies suggesting that they may not represent the general population. Longitudinal studies of population-based samples are needed. The study did not collect a comprehensive assessment of clinical lung diseases underscoring the need for further studies which can provide additional clinical characterization. White matter abnormalities and other structural changes of the brain were not included in these analyses and may explain additional variance of declining respiratory function (Taki et al., 2013). Since most of the post-mortem indices were obtained from different brain regions the findings may underestimate the association CNS pathologies located in brainstem and spinal cord locations which comprise the respiratory network.

## Conclusion

In this study, we assessed repeated measures of respiratory function, based on SPI and RMS in more than 1,400 community-dwelling older adults for up to 13 years. While there was considerable variability in the person-specific trajectories of change in respiratory function, nearly all participants exhibited some degree of progressive decline of both SPI and RMS and their rates of decline were moderately correlated. These data may have important consequences for interventions to decrease declining respiration in older adults. First, many older individuals may require interventions not only for impaired lung function, but are also likely to require care for concomitant respiratory muscle weakness. Second, the strong correlation between spirometric and RMS measures, lends support for clinical efforts to improve respiratory function through RMS training in addition to strategies to treat the causes of lung disease (Kim and Sapienza, 2005; Kulnik, 2015; Messaggi-Sartor et al., 2015).

Among those who died and underwent autopsy, indices of brain pathologies were associated with declining respiration. However, different brain pathologies were associated with the measures of declining SPI and RMS, underscoring the importance of assessing both constructs. Nigral neuronal loss was associated with the rate of change of SPI. In contrast, macroinfarcts and AD pathology were associated with declining RMS. These novel data suggest that age-related brain pathologies known to be associated with late-life cognitive and motor impairment are also associated with declining respiration in older adults. Linking respiratory decline with common brain pathologies provides a host of new targets and pathways that may lead to interventions for impaired respiration in older adults. For example, that the association of PD pathology with respiratory decline suggests that there may be a much larger number of older adults without clinical PD whose respiratory function may benefit from treatments developed for clinical PD or that aggressive primary preventive therapies for CVDs might slow the rate of respiratory decline in older adults without clinical strokes who may never report strokes.

## Future Directions

Further work is needed to determine if the association between brain pathology and respiratory decline is due to a third, latent variable which causes brain pathology and respiratory decline in older adults. If a latent variable is not identified, additional studies will be necessary to delineate the causal direction of the associations between brain pathology and respiratory decline during life, since primary pulmonary disease can cause impaired brain function. Answering these questions is essential before it will be possible to explicate the mechanisms underlying these associations.

The association between brain pathology and declining respiration only reduced the slope variance by a small percentage. This is similar to a prior study which examined other volitional motor performances, whose CNS pathways also extend beyond the brain (Buchman et al., 2014). The distributed neural networks which control respiration are complex and extend from the brain through specialized brainstem structures to the spinal cord to exit the CNS via peripheral nerve to peripheral muscle and pulmonary structures. In addition, these motor networks are modulated by sensory feedback circuitries. Further studies are needed to build on this study which focused on brain pathology. It will be necessary to collect post-mortem indices from the entire extent of the specialized structures which constitute the distributed respiratory network to fully explicate the pathologic basis underlying declining respiration in older adults.

## Author Contributions

All authors (AB, LY, BD, RW, VV, JS, DB) made substantial contributions to the conception and design of the work, acquisition, analysis or interpretation of the data. All contributed to drafting or revising the manuscript. All gave final approval to the version submitted for publication. All agree to be accountable for all aspects of the workin ensuring that questions related to the accuracy or integrity of any part of the work are appropriately investigated and resolved.

## Conflict of Interest Statement

The authors declare that the research was conducted in the absence of any commercial or financial relationships that could be construed as a potential conflict of interest.
